# The critical role of biochar to mitigate the adverse impacts of drought and salinity stress in plants

**DOI:** 10.3389/fpls.2023.1163451

**Published:** 2023-05-08

**Authors:** Yanfang Wu, Xiaodong Wang, Long Zhang, Yongjie Zheng, Xinliang Liu, Yueting Zhang

**Affiliations:** ^1^ Camphor Engineering Technology Research Center for National Forestry and Grassland Administration, Jiangxi Academy of Forestry, Nanchang, China; ^2^ College of Life Sciences, Nanjing Agricultural University, Nanjing, China

**Keywords:** antioxidants, biochar, drought, osmotic stress, salinity, soil organic matter

## Abstract

Drought stress (DS) is a potential abiotic stress that is substantially reducing crop productivity across the globe. Likewise, salinity stress (SS) is another serious abiotic stress that is also a major threat to global crop productivity. The rapid climate change increased the intensity of both stresses which pose a serious threat to global food security; therefore, it is urgently needed to tackle both stresses to ensure better crop production. Globally, different measures are being used to improve crop productivity under stress conditions. Among these measures, biochar (BC) has been widely used to improve soil health and promote crop yield under stress conditions. The application of BC improves soil organic matter, soil structure, soil aggregate stability, water and nutrient holding capacity, and the activity of both beneficial microbes and fungi, which leads to an appreciable increase in tolerance to both damaging and abiotic stresses. BC biochar protects membrane stability, improves water uptake, maintains nutrient homeostasis, and reduces reactive oxygen species production (ROS) through enhanced antioxidant activities, thereby substantially improving tolerance to both stresses. Moreover, BC-mediated improvements in soil properties also substantially improve photosynthetic activity, chlorophyll synthesis, gene expression, the activity of stress-responsive proteins, and maintain the osmolytes and hormonal balance, which in turn improve tolerance against osmotic and ionic stresses. In conclusion, BC could be a promising amendment to bring tolerance against both drought and salinity stresses. Therefore, in the present review, we have discussed various mechanisms through which BC improves drought and salt tolerance. This review will help readers to learn more about the role of biochar in causing drought and salinity stress in plants, and it will also provide new suggestions on how this current knowledge about biochar can be used to develop drought and salinity tolerance.

## Introduction

**Figure 1:**
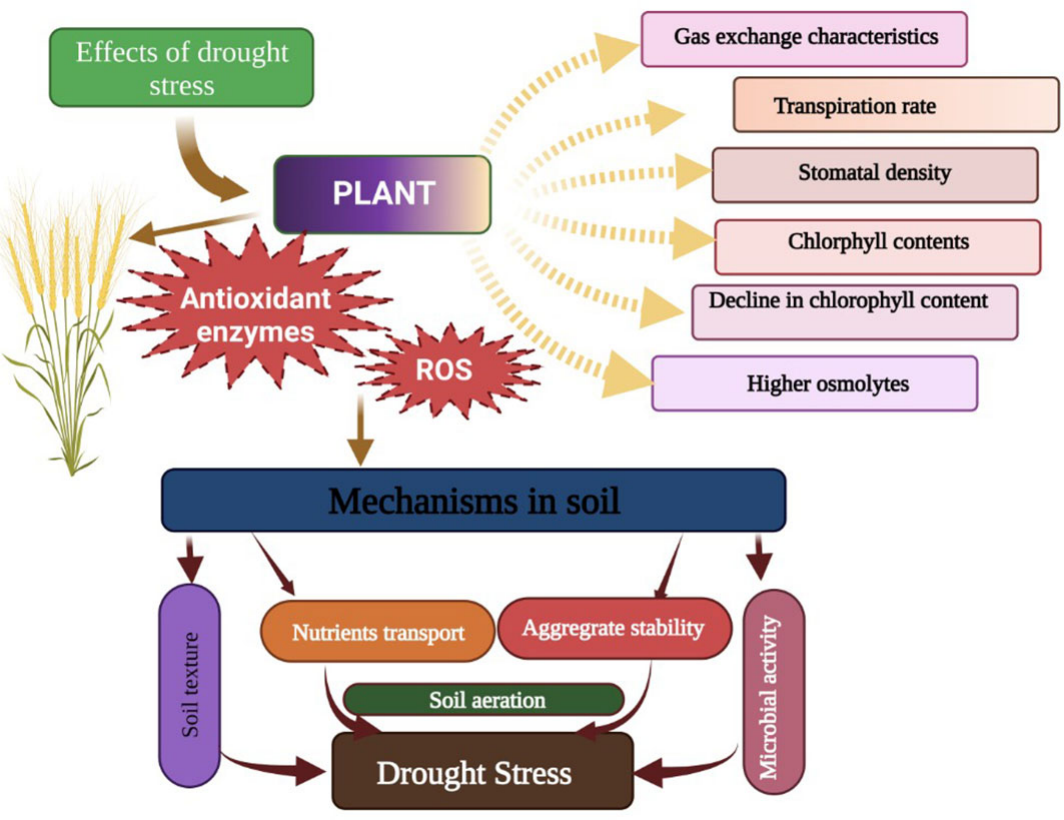
Effects of drought stress on plants. Drought stress disturbs the gas exchange characteristics, transpiration rate, stomata density, chlorophyll contents, soil microbial activities, soil aeration, and nutrients, thereby leadings to a significant reduction in plant growth.

Abiotic stresses are a serious threat to crop productivity and global food security. The intensity of abiotic stresses (drought, heat, heavy metals, and salinity) is continuously increasing, which is negatively affecting crop productivity (Hassan et al., 2021). Among these abiotic stresses, drought and salinity stress (SS) are serious abiotic stresses that are responsible for a substantial reduction in crop yields across the globe (Singh and Takhur, 2018). Drought stress (DS) is a serious abiotic stress responsible for a substantial reduction in crop productivity (Liang et al., 2020; Latif et al., 2022). Drought stress disturbs various functions from morphological levels to physiological and anatomical levels (**Figure 1**; Zhao et al., 2022). DS increases leaf senescence and decreases chlorophyll synthesis, which leads to a substantial decline in photosynthesis and crop productivity (Sunaina et al., 2019; Ma et al., 2021). DS also induces the overproduction of ROS (Vijayaraghavareddy et al., 2022), which damages proteins, lipids, DNA, and enzymatic reactions (Cui et al., 2017). Besides this, DS also negatively affects physiological processes and impacts agronomic traits, which cause a decline in grain productivity (Zhou et al., 2007). However, yield losses largely depend on the severity and duration of DS and plant species (Agarwal et al., 2016). Moreover, DS also causes stomata to close which decreases the conductance of stomata to the loss of water and negatively affects photosynthesis and transpiration rates (Mahmoud and Swaefy, 2020). Additionally, DS causes the destruction of enzymes and proteins along with a reduction in the synthesis of chlorophyll, which in turn causes a marked reduction in photosynthesis and, subsequently, plant productivity (Mahmoud et al., 2022a; Mahmoud et al., 2022b).

Salinity stress is continuously increasing globally, and it has been reported that 900 million hectares around the globe are salt-affected (Hernández-Herrera et al., 2022). By the end of 2050, half of the arable land will be damaged by SS (Denaxa et al., 2022) owing to the continuous accumulation of salts due to fertilization, salty irrigation, and climate change (Denaxa et al., 2022). Salinity stress inhibits the germination, growth, development, and productivity of plants in both dry and irrigated regions (Sultan et al., 2021). Salinity stress is an intense abiotic stress that negatively affects plant physiological and biochemical processes and leads to a serious reduction in growth and yield (Sofy et al., 2020).

Salinity stress limits germination, growth, and development by inducing osmotic stress and pseudo-drought stress (Parida and Das, 2005). The increased concentration of salts in the root zone under SS causes metabolic disorders, affects the photosynthetic efficiency, and assimilates production, which resultantly affects plant growth rate (Ghaderi et al., 2018; Mushtaq et al., 2022; Raza et al., 2022). Salinity stress also disrupts the ionic balance in cells and leads to excessive production of reactive oxygen species (ROS) (Hasanuzzaman et al, 2021), which causes oxidation of crucial molecules such as membranes, lipids, proteins, and carbohydrates (**Table 1**), alters redox homeostasis, and hinders the plant growth (Aref et al., 2016; Sultan et al., 2021). Moreover, SS also increases electrolyte leakage and MDA accumulation, and it also disturbs nutrient uptake, thereby reducing plant growth (Sultan et al., 2021). Therefore, as a matter of global food security, solutions must be put forth to reclaim and treat salt-affected soils to support better plant growth and productivity under SS conditions (Hernández-Herrera et al., 2022).

**Table 1:** Effect of drought stress on growth, physio-biochemical process, and antioxidant activities of various crops.

Crop species	Drought stress	Effects	References
Wheat	60% FC	The fresh and dry weight of shoot and roots chlorophyll contents total soluble protein and free amino acids decreased under DS.	Noreen et al. (2017)
Maize	75% FC	DS significantly reduced shoot weight (fresh and dry), root weight (fresh and dry), Chlorophyll (a, b, and total chl), and increased free proline, GB, H_2_O_2_, ASA and POD activity.	Shafiq et al. (2019)
Wheat	40% FC	DS reduced root fresh weight, root dry weight, Chl b and total chlorophyll, while increased EL, SOD, POD, CAT and MDA contents.	Amjad et al. (2021)
Sugar beat	30% FC for ten days at 30 days old stage seedling	DS resulted in a sharp increase in H_2_O_2_ and MDA and decrease in RWC, chlorophyll and carotenoids contents.	Islam et al. (2022)
Sunflower	30% FC	DS reduced the yield components, RWC, chlorophyll contents and increased proline and soluble sugars, phenylalanine ammonia lyase (PAL) and POD activities.	Ibrahim et al. (2016)
Maize	60% FC	DS increased MDA and H_2_O_2_ contents and increased CAT, POD and SOD activities. DS also triggered accumulation of soluble sugars, GB, proline, and phenolic contents.	Parveen et al. (2019)
Radish	70% FC	DS reduced growth and yield related traits along with increased in POD and SOD activities.	Noman et al. (2018)

Biochar (BC) has emerged as an excellent tool to improve crop productivity (Palviainen et al., 2020; Moragues Saitua et al., 2023) and tolerance to abiotic stresses (Tang et al., 2022). It has a high cation exchange capacity and an alkaline nature, which makes it an important amendment for the reclamation of salt-affected soils (Lehmann and Joseph, 2009; Lashari et al., 2013). The application of BC reduced the harmful impacts of SS by improving soil physiochemical and biological processes and Na leaching (Dahlawi et al., 2018). Biochar addition to salt-affected soils improves potassium (K^+^) uptake and reduces Na^+^ uptake, which in turn improves plant performance under SS (Drake et al., 2016; Usman et al., 2016). Recently, BC has also been identified as an important tool to improve crop productivity and water use efficiency (WUE) (Singh et al., 2019). The application of BC-enhanced nutrient uptake, carbon assimilation, and antioxidant activities, therefore, leads to an appreciable increase in plant growth under DS (Sorrenti et al., 2016; Wang et al., 2018; Wang et al., 2020). Moreover, BC also improves chlorophyll synthesis, WUE, and stomata conductance, thereby improving plant growth under DS (Paneque et al., 2016; Ramzani et al., 2017; Langeroodi et al., 2019; Haider et al., 2020). Further, BC application also improves soil physiochemical and biological properties that also induce favorable impacts on plant growth under DS (Agbna et al., 2017). Therefore, in this review, we have presented information on various mechanisms of BC in mitigating drought and salinity stresses in plants. We also identified the various research gaps that must be filled to realize the promising future of biochar as a soil amendment. This is the first detailed review of the role of BC in mitigating salinity and drought stress, and it will provide better insights into existing knowledge of BC in improving plant tolerance to both SS and DS.

## Why biochar is an important amendment

Biochar (BC) is a carbon-rich material that can be used as a soil conditioner to improve soil carbon sequestration and soil quality. Biochar is prepared from organic materials through a pyrolysis process (250–700°C; Rajakumar and Sankar, 2016), and it has various unique and special properties that make it an efficient, environment-friendly, and economical source of soil conditioner (Oliveira et al., 2017). Biochar is a porous and fine-grained material, and it has a similar appearance to charcoal; however, the only difference between the two is their utilitarian intention (Kapoor et al., 2022). The characteristics of BC depend on feedstock and pyrolysis conditions (Bird, 2015; Agegnehu et al., 2017). The pyrolysis temperature makes a difference in the properties of BC. For instance, Jindo et al. (2014) prepared the BC at different temperatures (400, 500, 600, 700, and 800°C), and they found that the BC obtained at 600°C has high recalcitrant characteristics as compared to the BC obtained at other temperatures (Kapoor et al., 2022). BC has a porous structure with many pores, which provides excellent habitat for soil microbes (bacteria, action-myocytes, and AMF) to colonize, grow, and reproduce, which in turn improves soil health and plant performance (Kapoor et al., 2022). Moreover, BC also improves soil nutrient holding capacity, water uptake efficiency, soil organic matter, and soil physiochemical and biological properties, which improve plant growth (Minamino et al., 2019; Ohtsuka et al., 2021; Kapoor et al., 2022; Qu et al., 2022).

## Biochar production and important feed stocks for biochar preparation

Biochar is produced through the pyrolysis process, which involves the heating of biomass in the complete or near absence of oxygen. During the pyrolysis process, oil, char, and gases are produced; however, processing conditions largely affect the quantity of these materials (Kapoor et al., 2022). The chemical composition of feedstock is reflected in the composition of BC, which also defines BC behavior, function, and fate in soil (Kapoor et al., 2022). Secondly, the extent of physio-chemical properties undergone by biomass during pyrolysis also depends on pyrolysis conditions like residence time and temperature (Verheijen et al., 2009). Globally, different materials, including wood, nut shells, husks, manures, and crop residues, are being used as feedstock to prepare the BC (González et al., 2009). Similarly, other feedstocks, including sewage sludge and municipal wastes, are also being used to prepare the BC; however, a risk is associated with the use of these materials owing to the presence of heavy metals (HM) in these feedstocks (Kapoor et al., 2022). The biomass with high mineral contents, like grasses, grain husks, and straw residue, produce BC rich in ash (Demirbas, 2004). Additionally, pyrolysis of wood-based feedstocks produces coarser and more resistant BC with carbon contents up to 80% (Winsley, 2007). Therefore, BC application could be an effective approach to improving soil health and plant productivity (Brown, 2009).

## Biochar a promising amendment to mitigate drought stress

Biochar has emerged as an excellent tool to mitigate the deleterious effects of drought stress (Siebielec et al., 2020). Biochar, being the black gold of agriculture, has received significant attention in recent times to offset the negative effects of DS (Zhang et al., 2019). The BC amendment increases SOC, soil moisture contents, water and nutrient uptake, CEC, and aggregate stability, which helps with drought tolerance (Zheng et al., 2018b; Odugbenro et al., 2020).

## Biochar maintains membrane stability and plant water relations under drought stress

Drought stress induces harmful effects on plasma membranes, and it also causes dehydration of the cytoplasm (**Figure 1**), which consequently increases electrolyte leakage and lipid peroxidation (**Table 2**, Hassan et al., 2021). The BC and chitosan addition enhanced DS tolerance in barley plants by decreasing EL and lipid peroxidation by improving membrane stability, RWC, and water pressure (Hafez et al., 2020). In another study, *Medicago ciliaris* plants grown under DS treated with BC showed a significant reduction in MDA concentration. Likewise, Yildirim et al. (2021) also found that BC decreases MDA accretion in *Brassica oleracea* by increasing the activity of antioxidant enzymes (Yildirim et al., 2021). There is a controversial role of BC on plant water status; for example, it was noted that BC improved the WUE and biomass production, but did not improve the RWC (**Table 2**). The assumption is that BC improved the plant nutrition status and increased the K uptake, which enhanced the stress resistance (Mannan et al., 2021).

**Table 2:** Effect of biochar on growth, and physiological attributes under drought stress.

Crop	Drought stress	Biochar application	Effects	References
Wheat	30% FC	37.18 g kg^−1^	BC significantly improved tillers, spike length, grains, grain weight, chlorophyll contents, stomatal conductance and RWC.	Haider et al. (2020)
Soybean	Pots were watered after 2 days	20 t ha^−1^	BC amendments improved seed vigor, germination percentage, shoot length, membrane stability index, chlorophyll and carotenoid contents and decreased sugar and proline contents.	Hafeez et al. (2017)
Wheat	Skip irrigation at tillering/grain formation	38 g kg^−1^	Biochar increased plant height, spike length, grain weight, photosynthetic activity, and grain yield.	Zaheer et al. (2021)
Chickpea	60% FC	3% (w/w)	BC improved root and shoot length, chlorophyll contents, RWC, and membrane stability index.	Hashem et al. (2019)
Eggplant	50% FC	500 g m^-2^	BC improved growth and yield components along with increase in Chl a and b contents and anti-oxidative activity.	Ebrahimi et al. (2021)
*Ehretia asperula*	DS was imposed by skipping irrigation.	15 t ha^−1^	BC increased in plant height; leaves/plant, REW and membrane stability.	Hoang et al. (2021)
Cabbage	50%FC	10%	Biochar amendment increased the RWC, photosynthetic activities, plant growth, nutrient uptake, and reduced MDA, H_2_O_2_, proline, and sucrose content.	Yildirim et al. (2021)

However, some authors noticed that BC application not only improved the WUE, but it also improved soil water holding capacity, and consequently plant water status (Laird et al., 2010; Licht and Smith, 2017). Likewise, another group of authors also noticed that BC amendment in sandy loam enhanced the plant available water (PAW) and improved RWC under DS (Aller et al., 2017). In another study, Licht and Smith (2017) found that BC application to frozen soil mitigated the adverse effect of DS by improving soil water contents, photosynthesis, leaf transpiration, and water under water stress conditions. Moreover, in maize plants, it was also reported that BC applications (2% and 3%) appreciably improved the leaf water potential and photosynthesis under DS (Ahmed et al., 2016). Haider et al. (2015) also found that BC application improved the RWC in poor sandy soil, whereas Lyu et al. (2016) revealed that BC addition strengthened antioxidant activities and plant water relations; however, they also found that BC response to DS varies according to plant species, soil, and BC type. In conclusion, BC application improves water uptake and RWC and thereby improves drought tolerance by increasing plant physiological functioning.

## Biochar improves nutrient uptake and maintain nutrient homeostasis under drought stress

Drought stress disturbs nutrient homeostasis and causes significant growth and yield losses. However, BC, being a promising soil amendment, improves nutrient homeostasis and leads to an appreciable increase in plant growth. For instance, BC applications (0.75% and 1.5%) improved the N uptake, which could be due to a BC-mediated increase in N retention in soil (Muhammed et al., 2020). Similarly, Ibrahim et al. (2020b) suggested that BC effectively works as a slow-release N fertilizer and improves plant performance under DS. In addition, BC-mediated increase in N uptake is linked with improved CEC, as soil with a higher CEC has a better ability to NH^4+^ and N utilization (Liang et al., 2014). Glaser et al. (2002) also noted that BC application improves Ca, Mg, and K concentrations in soil solution, which in turn increases the availability of nutrients under DS.

**Figure 2:**
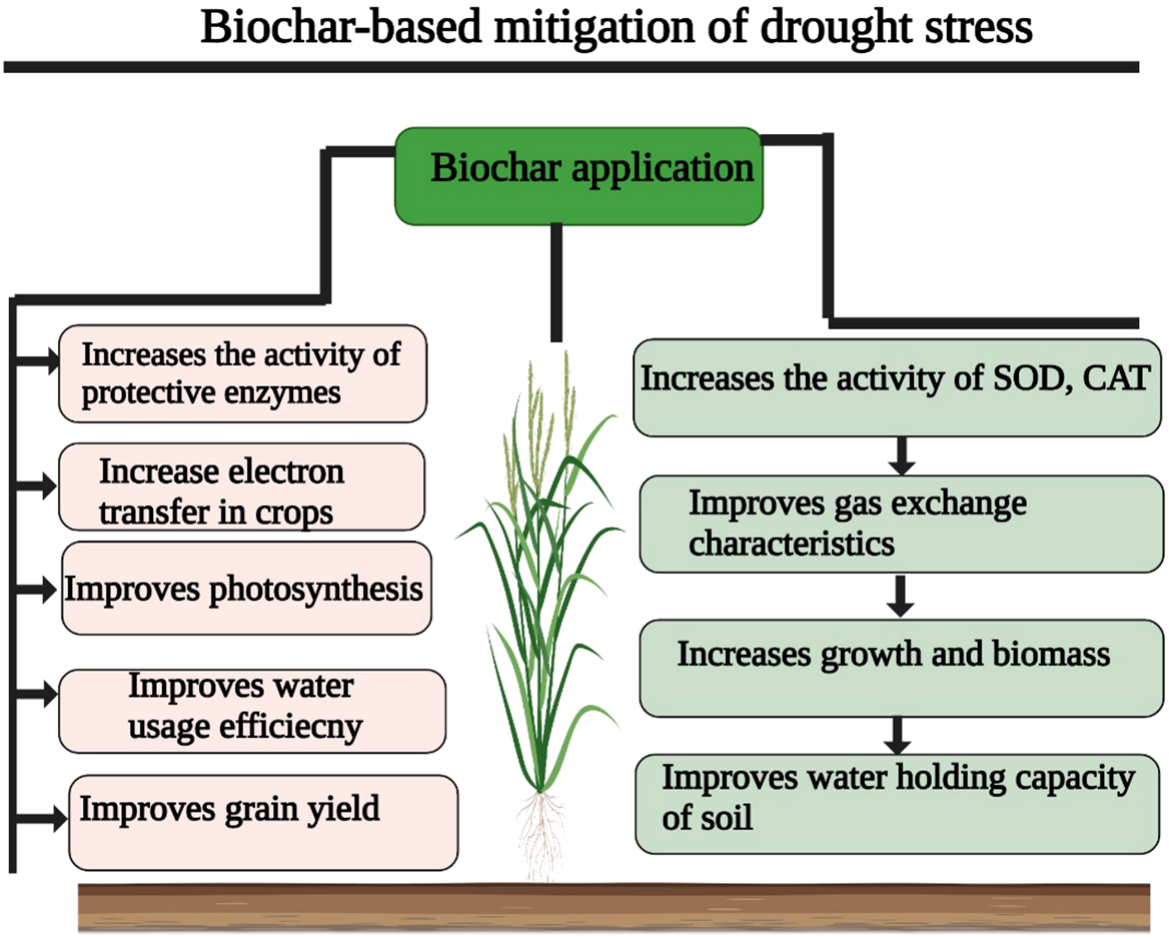
Biochar application increases antioxidant activities, membrane stability, photosynthesis, water use efficiency, gas exchange characteristics, water holding capacity, genes expression, and osmolytes accumulation and leads to a significant increase in drought tolerance.

Further, according to Van Zwieten et al. (2010), BC increases soil nutrient availability by affecting soil pH (**Figure 2**). In another study, it was found that BC applications (0.75% and 1.5%) improved the N uptake and mitigated the adverse effects of DS on peanuts by improving the N uptake (Zhang et al., 2021). Zoghi et al. (2019) found that BC application improves nutrient uptake, possibly by increasing soil WUC and WUE under DS conditions. Egamberdieva et al. (2017) reported that BC in combination with *Bradyrhizobium* substantially improved growth and NP uptake as compared to control conditions. Likewise, Liu et al. (2017) noted that BC (birch wood, 500°C) in combination with *Rhizophagus irregularis* increased the leaf area, N and P uptake, and WUE; however, BC in combination with *R. irregularis* had no significant impact on soil pH and root biomass under DS.

Şahin et al. (2016) found that BC enhanced N concentration, whereas Durukan et al. (2020) exposed the sugar beet plants to DS and BC. They found a higher concentration of P under 100% FC (field capacity) with 0.5% BC; however, P concentration was decreased with a parallel increase in BC application, which indicates that the rate of BC plays a significant role in nutrient uptake under DS. Langeroodi et al. (2019) treated the pumpkin plants with different DS levels (45%, 60%, 75%, and 90%) and BC rates (0, 5, 10, and 20 t ha^−1^). They found that Mg concentration was increasing with BC rate, and higher Mg concentration was noted under medium DS with 20 t ha^−1^. In another study, Poormansour et al. (2019) reported that increasing the rate of BC application (0%, 1.25%, 2.5%, 3.75%, and 5%) to faba bean plants under diverse irrigation levels (100%, 75%, and 50% of the water requirement) increased the Ca and Mg uptake and concentration in soil. To summarize, BC increased the CEC and nutrient uptake, which in turn improved drought tolerance by favoring a substantial increase in antioxidant activity and physiological functioning.

## Biochar protects photosynthetic apparatus and improves photosynthesis under drought stress

The devastating impact of DS on photosynthesis contributes to a reduction in chlorophyll synthesis, leaf area, and electron transport. It is well established that BC application improves the synthesis of growth-regulating substances, which improve plant functioning under DS (Manolikaki and Diamadopoulos, 2019). BC addition supports water deficit conditions by increasing chlorophyll contents and antioxidant activities (Lyu et al., 2016). Previously, it has been reported that BC amendment buffers the effects of DS on carbon assimilation and photosynthesis, which is linked with boosted chlorophyll synthesis and a less pronounced reduction in stomata conductance (Zainul et al., 2017; Wang et al., 2021).

Under DS conditions, BC application enhanced the WUE, which in turn improved the net photosynthetic rate and reduced non-stomatal limitations (Paneque et al., 2016). Likewise, BC application mitigated the adverse effect of DS by improving WUE, stomata conductance, and chlorophyll synthesis in cowpea, okra, and tomato plants (Farooq et al., 2021). Recently, it has also been reported that BC treatment increases productivity and reduces ROS damage by increasing chlorophyll synthesis and photosynthetic rate, and lowering electrolyte leakage (Gharred et al., 2022). Besides, this BC also brings ultra-structural changes to improve photosynthesis. For instance, BC application improved the stomata length, as well as the width and density of stomata, which significantly improved WUE and photosynthetic rate under DS conditions (Akhtar et al., 2014; Khan et al., 2021).

The application of BC increased the RWC, which improved the transpiration and photosynthetic rates as well as leaf osmotic potential under DS conditions (Haider et al., 2015). Kammann and Graber (2015) studied the impact of DS on drought tolerance in quinoa plants. They found that BC improved the soil properties, leaf water status, and photosynthesis and led to an appreciable increase in overall plant photosynthetic efficiency and assimilation production under water-scarce conditions (Haider et al., 2015; Ahmed et al., 2016; Lyu et al., 2016). The BC application to poor sandy soil improves plant water relations, photosynthesis, and growth by reducing toxic effects, or ROS (Haider et al., 2015; **Table 3**). In another study, Lyu et al. (2016) noted that BC application improves electron transport and enzymatic activities, by reducing the damaging effects of DS on the photosynthetic apparatus. In essence, BC protects the photosynthetic apparatus from drought-induced oxidative stress and increases the synthesis of photosynthetic pigments, thus resulting in a significant increase in photosynthesis under drought stress.

**Table 3:** Effect of biochar on various oxidative stress markers under drought stress.

Crop	Drought stress	BC application	Effects	References
Tall reed	40% FC	0.75%	BC application significantly lowered MDA accumulation due to increased antioxidant activities.	Abideen et al. (2020)
Rapeseed	35% FC	30 t ha^−1^	BC reduced H_2_O_2_, MDA and electrolyte leakage owing to increased antioxidant activities.	Khan et al. (2021)
Pumpkin	60% FC	20 t ha^−1^	Biochar ameliorated the adverse effect of ROS and improved membrane stability index.	Langeroodi et al. (2019)
Pyrus ussuriensis Maxim.	80%	9 t ha^−1^	BC protected membrane by reducing the activity of MDA and H_2_O_2_.	Lyu et al. (2016)
Wheat	75% FC	2%	Biochar enhanced membrane stability by reducing H_2_O_2_ and MDA.	Lalarukh et al. (2022)
Ryegrass	25% FC	10%	BC improved membrane stability by decreasing by H_2_O_2_ contents and increasing antioxidant activities.	Safari et al. (2022)
Sainfoin	64% FC	1.7%	Biochar enhanced leaf RWC and membrane stability index by reducing MDA and H_2_O_2_ accumulation.	Roy et al. (2022)

## Biochar maintains osmolytes accumulation and hormonal balance under drought stress

Osmolytes play an indispensable role against DS; however, it has been reported that DS disturbs the hormonal balance and osmolyte accumulation. Proline produced under stress conditions works as an ROS scavenger as well as for osmotic adjustment. For instance, DS in *M. ciliaris* leaves significantly increased the proline accumulation; however, findings of Yildirim et al., 2021 showed that BC-treated plants reduced the proline contents, possibly due to less ROS production and reduced oxidative and osmotic stresses in BC-amended plants. In another study, BC in combination with chitosan markedly reduced the soluble sugars, sucrose, and starch contents in stressed and controlled barley plants (Hafez et al., 2020). In a study, Gullap et al. (2022) found that BC application and irrigation levels significantly affected the ABA, IAA, and GA contents of soybean plants. These authors noted that IAA and GA contents were reduced, whereas ABA contents were increased under DS. Nonetheless, BC also increased the IAA and GA and decreased the ABA contents of soybean-treated plants (Gullap et al., 2022). Thus, BC maintains hormones and osmolyte accumulation, which protect the plants from drought-induced oxidative stress and substantially improve drought tolerance.

## Biochar improves antioxidant systems and detoxify ROS under drought stress

Drought stress induces oxidative stress by increasing ROS that damage the major molecules of plants. For instance, an increase in SOD activity in the water-stressed plant was linked with improved photoprotection and an increase in membrane stability (Gharred et al., 2022). BC application strengthens antioxidant activities; for instance, an increase in SOD and APX activity in control and water-stressed plants was observed with BC application (Gharred et al., 2022). BC application buffers the toxic effects of DS on the plant photosynthetic apparatus by regulating electron transport and antioxidant activity (Chaves et al., 2009). Foyer et al. (2009) found an increase in the AsA/DHAsA ratio and SOD, APX, GPX, and GR activities at DS, which was not sufficient to counter the effects of DS (**Table 4**). However, BC application (2%) under DS increased the AsA/DHAsA ratio, SOD, APX, GPX, and GR activities and encountered the toxic effects of DS through ROS scavenging (Foyer et al., 2009).

**Table 4:** Effect of biochar on various osmolyte and antioxidant activities under salinity stress.

Crop	Salinity stress	BC application	Effects	References
Common bean	12 dS m^-1^ NaCl	20% total pot mass	BC increased magnitudes of proline, glycine betaine, soluble sugar, and TSP and increased APX and POD activities.	Farhangi-Abriz and Torabian (2017)
Cowpea	10 dS m^−1^	25 g per pot	Biochar application reduced the MDA content and antioxidant activity under SS.	Farooq et al. (2020)
Soybean	80 mM NaCl	3 g biochar	Biochar boosted the expression of CAT, APX, POD and SOD and reduced the salinity-induced increase in the NaH_2_O_2_ and MDA level.	Mehmood et al. (2020)
Jute	150 mM NaCl	2 g kg^−1^ soil	BC stimulated both non-enzymatic (e.g., ascorbate and glutathione) and enzymatic (e.g., APX, POD, and SOD) activities.	Hasanuzzaman et al, (2021)
Maize	150 mM NaCl	5%	BC application reduced sodium content, improved proline contents and POD under SS.	Fazal and Bano (2016)
Faba bean	1,500–3,000 ppm NaCl	15 t ha^−1^	BC decreased level of MDA, EL, O_2_^-^, and H_2_O_2_, through increased CAT, SOD, GR and POD activities.	El Nahhas et al. (2021)
Wheat	100 mM NaCl	5%	BC application increased proline, sugars and GB and activities of SOD, CAT, APX, DHAR, MDHAR and GR.	Alharbi and Alaklabi (2022)

Khan et al. (2021) found that BC application markedly improved the TSS and TSP contents, while BC proline contents indicated that BC could reduce the harmful effects of DS (Khan et al., 2021). Moreover, it was observed that BC in combination with AMF enhanced drought tolerance by improving osmotic adjustments, hormonal balance, and antioxidant activity (Mickan et al., 2016). Biochar addition to soil improves CAT, POD, and SOD activity (**Table 5**) by improving plant metabolic functioning, cell growth, and reducing ROS production, which results in substantial improvement in plant performance under DS (Zulfiqar et al., 2022). Additionally, barley plants treated with BC and chitosan under DS showed a marked improvement in CAT, POD, and GR activities, which resultantly reduced the drought-induced oxidative damage on barley plants (Hafez et al., 2020). In conclusion, a BC-mediated increase in antioxidant activities reduces ROS and protects the plants from the deleterious impacts of drought stress, therefore improving plant growth under drought stress.

**Table 5:** Effect of biochar on accumulation of various osmolyte and antioxidant activities under drought stress.

Crop	Drought stress	Biochar application	Effects	References
Rapeseed	35% FC	30 t ha^−1^	BC application increased SOD, POD, CAT, ASA, and GSH, proline, TSS and TSP accumulation.	Khan et al. (2021)
Pumpkin	60% FC	20 t ha^−1^	BC ameliorated the adverse effect of ROS, by increasing activity of SOD, POD, CAT, PPO, and APX and proline accumulation.	Langeroodi et al. (2019)
Ryegrass	25% FC	10% (w/w)	BC improved membrane stability, proline accumulation, and increased APX, POD and SOD activities.	Safari et al. (2022)
Sainfoin	64% FC	1.7%	Biochar decreased the content of soluble sugar (SS) and the activities of SOD, CAT, POD, APX and decreased MDA, H_2_O_2_ and proline contents.	Roy et al. (2022)
Wheat	75% FC	2%	Biochar upraised the levels proline, flavonoids, anthocyanin, phenolics, ascorbic acid, protein, glycine betaine and APX, POD and SOD.	Lalarukh et al. (2022)

## Biochar improves genes expression and stress responsive proteins under drought stress

BC application also improves gene expression to induce drought stress in plants. For instance, plants showed increased expression of CAT, APX, and Mn-SOD genes under 50% FC as compared to 75% and 100% FC; nonetheless, BC and vermin-compost applications reduced the expression level of CAT, APX, and MnSOD genes under all irrigation levels (Hafez et al., 2021). Conversely, BC application increased the expression of all these genes under DS (Racioppi et al., 2019). BC application also activates the auxin-responsive growth-promoting pathway, which stimulates the germination and growth of wheat plants treated with BC (Vissenberg et al., 2005). Xyloglucan endotransglucosylase/hydrolase (XTH) genes are involved in controlling the extensibility of the cell wall during plant growth stimulated by GAs and IAA (Sánchez-Rodríguez et al., 2010). The application of BC increases the expression of the XTH gene in the *Saragolla* cultivar, which stimulates the plant synthesis of GAs after BC treatment (Racioppi et al., 2019). Viger et al. (2015) found that modifications in soil pH and increase in K+ availability in BC-treated soil activate Ca^2+^ and ROS-mediated cell signaling, which in turn stimulate the IAA and BR growth-promoting pathways.

## Biochar nutrition improves plant growth, yield, and quality under drought stress

Drought stress reduces the growth of plants by decreasing photosynthesis, nutrient uptake, and increasing ROS production. However, BC application improves growth and biomass production by improving plant nutrition, antioxidant activities, and osmolytes accumulation (Gharred et al., 2022). BC application increased the leaf area of okra and maize plants (Batool et al., 2015; Haider et al., 2015) under DS and it also increased the biomass of wheat grown in the semi-arid Mediterranean (Olmo et al., 2014). BC-mediated increases in plant leaf area do good and constant supply of nutrients to plants by alleviating DS (Laird et al., 2010; Zhang et al., 2021).

Plants use different mechanisms including signaling pathways, gene expression, and accumulation of proteins and enzymes to cope with DS. It has been reported that DS increased the protein of *M. ciliaris* (Gharred et al., 2022) and they were further increased by the application of BC. BC application also increased the vegetative growth, seed production, and quality of sunflower plants grown under DS (Paneque et al., 2016). Likewise, another group of authors also found that BC application improved the growth, yield, and quality of tomato and rapeseed plants under DS (Bamminger et al., 2016; Agbna et al., 2017). Haider et al. (2015) noted that BC could improve the growth of water stresses plants by increasing soil-plant water relationships and photosynthesis. Egamberdieva et al. (2017) found that BC with *Bradyrhizobium* showed a significant increase in growth and biomass production and N and P concentration in lupin plants.

In another study, it was found that BC application (0–30 t ha^−1^) improved biomass and yield while BC application at the rate of 60 t ha^-1^ had adverse impacts and it negatively affected the rapeseed growth and seed production under DS. Similarly, BC application (0–30 t ha^−1^) improved the biomass, pods/plant, and 1,000 seed weight by 56%, 26%, and 15% and control conditions while BC application improves biomass, pods/plant, and 1,000 seed weight by 23%, 32% and 21% in drought conditions (Khan et al., 2021). DS also negatively affected the oil and protein contents; however, BC treatment led to a marked improvement in oil and protein contents under DS (Khan et al., 2021). Drought also increased the erucic acid contents, while BC application significantly decreased the erucic acid contents. Drought stress also led to a significant decrease in oleic acid; nonetheless, biochar appreciably improved oleic acid concentration under DS conditions. Similarly, compared to control conditions BC application (0–30 t ha^−1^) also showed a significant increase in linoleic acid under normal and DS conditions (Khan et al., 2021). Thus, the BC-mediated increase in growth and yield is linked with an increase in photosynthetic performance, antioxidant activities, gene expression, and osmolyte accumulation.

## Biochar improves soil properties to induce drought tolerance

Biochar has emerged as an excellent tool to improve soil health and crop productivity. It has been reported that BC improves soil physical properties, including soil density, soil moisture content, and aggregate stability under DS (Ye et al., 2016; Bamminger et al., 2016; Zhang et al., 2017). Zhang et al. (2017) reported that BC improved the soil properties and abundance of bacteria, which contributed to a substantial increase in the stress tolerance ability of tobacco plants. Likewise, other authors also noted that BC application improved soil bulk density, WHC, and water retention, which led to a significant increase in drought tolerance (Abel et al., 2013; Liang et al., 2014). Moreover, BC also improves aggregate stability in coarse-textured soils and soil WHC; both factors play a critical role in soil plant growth (Lehmann et al., 2011; Foster et al., 2016).

BC-mediated increase in soil WHC is due to the porous structure of BC and the higher CEC of BC (Laghari et al., 2016). Soil microbial biomass (SMB) plays an imperative role in OM decomposition. Higher SMB improves nutrient availability and soil fertility, and it also works as the linkage between the source and sink of soil nutrients (Marschner et al., 2015). Drought stress induces osmotic stress, which causes microbial death and a reduction in SMB (Sanaullah et al., 2011). Drought-mediated decrease in SMB decreases the decomposition of OM under DS Hailegnaw et al. (2019); however, BC has been reported to increase microbial activity, OM, and nutrient contents, therefore improving the SMB, which in turn improves soil fertility and plant growth (Cornelissen et al., 2018). Moreover, BC also increases soil organic carbon, which increases soil microflora and soil enzymatic activities that positively affect plant performance (Rahman et al., 2018; Rahman et al., 2021). DS imposes negative effects on soil biological properties, and BC substantially offsets these negative impacts and improves soil biochemical properties. For instance, BC application (38 g kg^−1^) appreciably improved the soil P (18.72%), K (7.44%), soil carbon (11.86%), nitrogen mineralization (16.35%), and soil respiration (6.37%), which in turn increased the soil microbial activities compared to the control and lower rates (28 g kg^−1^) of BC application (Zaheer et al., 2021). Therefore, a BC-mediated increase in OM, CEC, WHC, water retention, and bulk improves drought stress by bringing about favorable changes in plant functioning.

## How does biochar improve salinity tolerance?

Soil salinity represents the most critical constraint to crop productivity and global food security (Farouk et al., 2020; Sofy et al., 2020). Soil salinization is continuously increasing, which is posing a serious threat to crop productivity and food security (FAO, 2017). The area under soil salinity is significantly increasing, and salinity stress causes 27.2 billion USD annual losses in irrigated agriculture (Munns and Gilliham, 2015). It has been documented that 20% of cultivated and 33% of irrigated land globally is affected by salinity stress (Machado and Serralheiro, 2017). Further, soil salinized area is increasing at a rate of 10% annually owing to anthropogenic activities, which pose a serious threat to food productivity (Jamil et al., 2011; Dadshani et al., 2019). Biochar is a carbon-rich product used as an important soil conditioner to improve soil quality and plant performance (Ali et al., 2021). Further, BC application brought favorable changes in soil and plant functioning to improve SS tolerance (Ibrahim et al., 2020; Ali et al., 2021). The various mechanisms by which BC improves salinity tolerance are presented below.

## Biochar maintains membrane stability and plant water relations under salinity stress

Salinity stress negatively affects cellular membranes, and it damages the membranes by increasing MDA accumulation (Guo et al., 2019). The membrane damage due to SS also leads to the loss of important solutes (Mata-Pérez et al., 2015). The concentration and composition of fatty acids and lipids have a strong impact on membrane functioning, stability, and fluidity. For instance, unsaturated fatty acids play a critical role in membrane protein activity and membrane protection (Mikami and Murata, 2003). The application of BC has been reported to increase the concentration of unsaturated fatty acids, which in turn improves the membrane stability under SS (Ndiate et al., 2021). The application of BC also improves antioxidant activities (APX, CAT, POD, SOD, and GR), which also protects the membranes from the damaging effects of SS (Kim et al., 2016).

**Figure 3:**
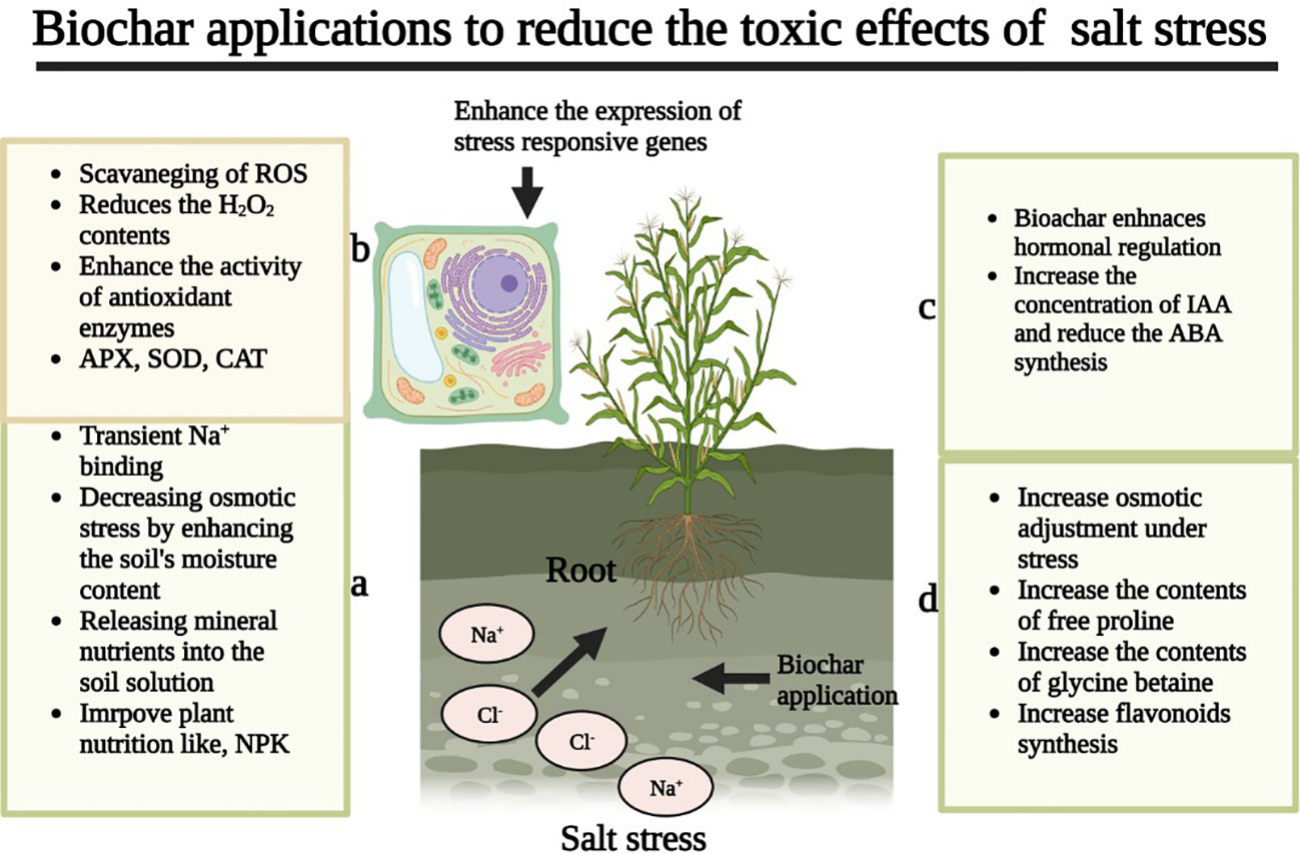
Biochar reduces ROS production, and increaseds membrane stability, antioxidant activities, osmolytes accumulation, gas exchange characteristics, soil properties, and nutrient uptake, and decreases the accumulation of toxic ions, thereby improve improving salinity tolerance.

Lashari et al. (2015) noted that the application of manure and BC application appreciably improved the membrane stability by decreasing MDA concentration (**Figure 3**). The application of BC also reduced electrolyte leakage and increased the relative water content (RWC) by making membranes stronger (**Table 6**) and protecting membranes from the toxic effects of Na^+^ (Ran et al., 2020). In another study, it was observed that BC addition improved membrane integrity by improving the concentration of unsaturated fatty acids and increasing the activities of antioxidant enzymes, which protect membranes from the damaging effects of oxidative stress (Ndiate et al., 2022). Biochar application also improves the leaf water status and protects the plants from the damaging effects of SS. For instance, it has been recorded that BC improved the leaf water status of rice at the heading and grain-filling stages as compared to no BC application (Ran et al., 2020). These authors further stated that BC application prevents membrane damage by decreasing Na^+^ content and increasing K+, which therefore improves the leaf water status under SS (Ran et al., 2020). In fact, BC improves the leaf water status by increasing K concentration, as K is considered an important osmoprotectant in plant tissues (Ran et al., 2020). The leaf RWC of plants is improved after BC application, resulting in an increase in WUE efficiency by plants (Naeem et al., 2017; Shabbir et al., 2021).

**Table 6:** Effect of salinity stress on growth, physio-biochemical process, and antioxidant activities of various crops.

Crop species	Salinity stress	Effects	References
Pea	100 mM NaCl	SS reduced shoot length, leaves/plant, days to flowering and chlorophyll contents and increased, proline contents, antioxidant activity and accumulation of Na^+^.	Ishrat et al. (2022)
Wheat	250 mM NaCl	SS reduced plant height, leaf area, biomass, grain weight, grain yield, chlorophyll, carotenoids, net photosynthetic rate, transpiration and stomatal conductance and increased antioxidants (SOD, POD, CAT) activity.	Desoky et al. (2021)
Water Dropwort	200 mM NaCl	The growth parameters, relative water contents, chlorophyll contents and Na^+^ accumulation was increased under SS.	Kumar et al. (2021)
Sweet pepper	4000 ppm	SS reduced chl a and b contents, RWC (%) while increased leakage, proline contents and activity of CAT and POD.	Abdelaal et al. (2019)
Tomato	100 mM NaCl	SS caused a reduction in growth parameters, photosynthetic pigments (Chl a and b), soluble sugars, soluble proteins, and K^+^ content. However, SS increased concentration of proline, MDA and H_2_O_2_.	Attia et al. (2021)
Cumin	100 mM NaCl	SS reduced germination index, plant biomass, and chlorophyll contents, however, SS increased, leucine, glycine, proline, H_2_O_2_, and MDA contents.	Pandey et al. (2015)
Wheat	200 mM NaCl	SS decreased K^+^ contents and K^+^/Na^+^ ratio, root and shoot length, relative growth rate chlorophyll fluorescence, and chlorophyll contents.	Saddiq et al. (2021)
Lettuce	100 mM NaCl	SS significantly reduced the leaf area, root and shoot growth and photosynthetic efficiency.	Al-Maskri et al. (2010)

Other authors also found that BC is an excellent strategy to improve the leaf RWC under SS. The application of BC improves water uptake and maintains the osmotic potential, which in turn improves the leaf RWC of plants growing under SS (Torabian et al., 2018; Soliman et al., 2022). Aquaporins play an imperative role in the transportation of water; for instance, PIPs not only improve the uptake and transportation of water to plant leaves and roots, but they also increase CO_2_ diffusion and ensure an abundant supply of substrates for photosynthesis as well as carbohydrate assimilation (Groszmann et al., 2017; Cui et al., 2021; Saibi, 2021). BC application increased carbon assimilation and upregulated AQP genes, which improved water uptake and leaf water status (Kayum et al., 2017). BC application has been reported to improve the activity of water transporter genes and improve the water-holding capacity of the soil, thereby improving the leaf water status under SS (Shashi et al., 2018; Soliman et al., 2022). Thus, BC reduces ROS, which ensures membrane protection and maintains better plant water relations under SS.

## Biochar improves nutrient uptake and maintain nutrient homeostasis under salinity stress

Soil salinity negatively affects the accumulation and uptake of various macro and micronutrients. For instance, it was reported that salt-affected plants contained 35%, 61%, 59%, 12%, and 11% lower N, P, K, Ca, and Mg in leaf tissues as compared to the control treatment (Soliman et al., 2022). The application of BC promoted the uptake of nutrients under controlled and SS conditions. BC application improves Ca^2+^ content, which improves SS tolerance through modification of cellular signaling pathways (Qin et al., 2019). As a result, BC application increased the K^+^/Na^+^ ratio and Ca^2+^ by the Ca^2+^- dependent SOS pathway (Soliman et al., 2022). In another study, it was reported that BC application improved P and Mn concentrations in lettuce plants (Hammer et al., 2015).

In another study, it was reported that BC increased tissue P contents in a dose-dependent way (Kim et al., 2016), whereas Usman et al. (2016) also found that BC substantially increased the P, K, Fe, Mn, Zn, and Cu concentrations (**Table 7**) in tomato plants growing under SS. Nonetheless, wheat straw-based BC increased P precipitation and reduced the P concentration in plants growing under sodic soils (Xu et al., 2016). Similarly, Na^+^ uptake by plants was also reduced after BC application (Hammer et al., 2015), which indicates that ionic homeostasis in plants is improved after the application of BC (Akhtar et al., 2015a; Mansoor et al., 2021). Hence, BC application is an effective approach to reduce Na^+^ absorption and increase plant growth in saline soils (Mansoor et al., 2021).

**Table 7:** Effect of biochar on growth, and physiological attributes under salinity stress.

Crop	Salinity stress	BC application	Effects	References
Rice	25 mM NaCl	3%	BC application increased plant growth, shoot biomass, tiller/plant, chlorophyll (a and b), transpiration rate, WUE, and concentration of K^+^.	Hafeez et al. (2019)
Wheat	100 mM NaCl	150 g	BC improved the plant height, shoot fresh biomass, root fresh biomass, and partially increased enzymatic activity and photosynthetic pigments.	Ndiate et al. (2022)
Spinach	150 mM NaCl	1%	The morphological parameters membrane stability index and RWC (%), nutrient (N, P, K) uptake was improved after BC application.	Sofy et al. (2021)
Cowpea	10 dS m^−1^	25 g	BC improved seedling emergence, ionic homeostasis, and redox balance, sugar metabolism, and chlorophyll synthesis.	Farooq et al. (2020)
Tomato	100 mM NaCl	10% v/v	Application of biochar augmented the plant height, leaf area, fresh and dry weight of shoot and root, RWC and chlorophyll contents.	Kul et al. (2021)
Wheat	150 mM NaCl	2%	Biochar application improved germination, seedling vigor index (SVI), and leaf water potential under SS.	Kanwal et al. (2018)
Wheat	100 mM NaCl	1%	Biochar application significantly enhanced physiological parameters along with shoot dry weight, chlorophyll a and b concentrations by decreasing MDA contents.	Khan et al. (2022)

The application of BC also leads to a slight increase in the EC of both normal and sodic soils, and an increase in this EC is linked with the release of various nutrients (Ca, K, and Mg) after the application of BC (Mehdizadeh et al., 2020). The higher quantity of Ca and mg released by BC displaces Na^+^ on soil exchange sites, which reduces the availability of Na^+^ for plants (Huang et al., 2019). The addition of BC hinders the uptake and accumulation of Na^+^ owing to the fact BC has a higher surface area, CEC, and porosity. Besides this, BC also has a high adsorption capacity, which reduces the negative effects of SS by increasing Na^+^ adsorption and increasing the release of beneficial nutrients (Ca, Mg, and K) (Iqbal et al., 2019). Further BC also improved the K/Na^+^ ratio and increased the water holding capacity, plant available water, and WUE, which reduced salinity-induced osmotic stress (Akhtar et al., 2015b; Naeem et al., 2017). The studies indicate that BC application increases the uptake of minerals under saline soils; nonetheless, more research is required to understand the mechanism of BC-mediated increase in nutrient uptake.

## Biochar protects photosynthetic apparatus and improves photosynthesis under salinity stress

Salinity stress predominantly decreases RWC and chlorophyll synthesis and leads to a substantial reduction in photosynthesis (Manan et al., 2016). BC application has been reported to increase the chlorophyll contents owing to an increase in N (Akhtar et al., 2015a). Under SS, BC has been shown to improve stomata conductance and the synthesis of chlorophyll and lead to an increase in photosynthetic efficiency (Feng et al., 2018; Shabbir et al., 2021). The application of BC to saline soils improves stomata density and stomata conductance, which improve leaf gas exchange characteristics, resulting in substantial increase in photosynthetic efficiency under SS (Thomas et al., 2013; Akhtar et al., 2015a). Biochar-mediated improvement in photosynthetic pigments is linked with nutrient uptake and availability (K, P, Mg, Ca, and S) and improvements in the physiochemical and biological properties of soil (Farhangi-Abriz and Torabian, 2018). The plants growing under SS exhibited more damage to photosynthetic pigments (Kaya et al., 2018); however, BC application appreciably improved the antioxidant activities, which prevent the oxidative damage to photosynthetic pigments and photosynthetic apparatus of plants growing under SS (Rasheed et al., 2019).

Biochar addition leads to a significant increase in chlorophyll and carotenoid contents under normal and stressful conditions, leading to the maintenance of greenish leaves (Ran et al., 2020). The increased nutrient uptake and assimilation in BC-treated soils improved the enzymatic activity, chlorophyll synthesis, photosynthesis, and tolerance against stress conditions (Soliman et al., 2022). The impact of BC on chlorophyll and carotenoid contents under SS is linked with accelerated antioxidant activity and the building of antioxidant activity. BC supplementation also provokes Mg^2+^ uptake, which is considered a building block in the synthesis of chlorophyll (Farouk and Al-Huqail, 2022). Moreover, BC also positively improves transpiration and stomata conductance, which leads to a substantial improvement in photosynthetic efficiency under SS as compared to no BC application (Akhtar et al., 2015b; Ghassemi-Golezani and Farhangi-Abriz, 2021). The deficiency of K affects chlorophyll fluorescence through an increase in thermal dissipation and a reduction in the efficiency of electron transport. The reduction in electron transport efficiency decreases NADPH and ATP concentrations, which decreases the activity of ATPase and increases NADP reduction under SS conditions (Ghassemi-Golezani and Rahimzadeh, 2022). BC application decreases the rate of NADP reduction through negative feedback from increasing NADPH in the plant. BC treatments provide more energy (NADPH, ATP) for photosynthetic activities by increasing the Hill reaction and decreasing the rate of NADP reduction, thereby increasing photosynthesis under SS (Lu et al., 2020). ATPases are considered as essential enzymes for the formation of adenosine diphosphate (ADP) and carbon assimilation during photosynthesis (Lu et al., 2020). BC application increases the activity of the Hill reaction, the functional integrity of PS-II, and electron transport efficiency, which is useful to decrease ROS production and increase plant photosynthetic efficiency (Ghassemi-Golezani and Rahimzadeh, 2022). Thus, BC-mediated increase in photosynthesis is linked to better synthesis of photosynthetic pigments, increased nutrient uptake, and improvements in soil physicochemical properties.

## Biochar strengthens antioxidant systems and detoxify ROS under salinity stress

Salt stress induces the excessive production of ROS that impose devastating impacts on plants’ membranes, proteins, and lipids (Abbas et al., 2022). BC treatment improved redox homeostasis and prevented the overproduction of ROS. For instance, it was recorded that BC application reduced the H^2^O^2^ and TBARS concentration at both levels of BC (1% and 2%) in saline-sodic soil. However, the application of 2% BC markedly reduced the H^2^O^2^ concentration (**Table 8**) as compared to 1%, which indicates that the rate of BC has a strong influence on mitigating salinity-induced oxidative stress (Abbas et al., 2022). BC application enhanced antioxidant activities (CAT, POD, and SOD; **Table 4**); however, this increase was sufficient to reduce the toxic effects of ROS (Abbas et al., 2021). The improved function of the AsA–GSH cycle prevents H^2^O^2^-mediated oxidative impacts by maintaining a redox balance, which protects the metabolic pathways, photosynthesis, and enzyme functions (Alam et al., 2020).

**Table 8:** Effect of biochar on various oxidative stress markers under salinity stress.

Crop	Salinity stress	BC application	Effects	References
Borage	50 mM NaCl	5%	The addition of biochar mitigated the effect of salinity by reducing Cl^−^, Na^+^, and Na^+^-translocation, and H_2_O_2_ and MDA accumulation.	Farouk and Al-Huqail (2022)
Spinach	150 mM NaCl	1%	Biochar reduced electrolyte leakage, lipid peroxidation, H_2_O_2_, OH contents, MDA and ROS production SS.	Sofy et al. (2021)
Tomato	100 mM NaCl	10%	BC improved membrane leakage by increasing SOD, POD, CAT activity.	Kul et al. (2021)
Wheat	150 mM NaCl	2%	Biochar application improved osmotic potential increasing membrane stability, proline content, and soluble sugar percentage.	Kanwal et al. (2018)
Quinoa	30 mM NaAsO_2_	2%	Biochar ameliorated the oxidative stress by increasing the activities of antioxidant enzymes (SOD, POD, CAT).	Shabbir et al. (2021)
Rapeseed	12 dS m^−1^ NaCl	30 g biochar per kg soil	Biochar protected the membrane by decreasing lipid peroxidation, H_2_O_2_, and MDA accumulation due to increase in SOD, POD, CAT, POX, phenols, and flavonoids.	Ghassemi-Golezani and Abdoli (2022)
Wheat	EC: 4.3 dS m^−1^	1 kg m^−2^	The application of BC reduced the oxidative stress by increasing CAT, APX, and Mn-SOD genes.	Hafez et al. (2021)
Sorghum	12.6 dS m^−1^	5%	BC protected the membrane stability by decreasing MDA accumulation owing to increased SOD, POD and CAT.	Hussien Ibrahim et al. (2020)

BC supplementation improves AsA–GSH activity and the activities of other antioxidant enzymes that prevent the production and accumulation of ROS (Soliman et al., 2022). Likewise, Rasheed et al (2019) also noted that BC application decreased ROS production and consequently reduced lipid peroxidation with the help of enhanced antioxidant enzyme activity. However, some authors found a reduction in antioxidant activities (APX and GR) by using BC, possibly due to less uptake of Na^+^ in BC-treated plants (Kim et al., 2016). Recently, it has also been observed that BC application reduced the activity of antioxidant enzymes and oxidative stress in bean seedlings compared to control (Farhangi-Abriz and Torabian, 2017). The application of BC improved the AsA and GSH contents and reduced the DHA and GSSG content, therefore, improving the capability for detoxification of ROS under SS (Hasanuzzaman et al., 2021; Turan, 2021).

The increase in APX activity was also reported in soybean-treated plants following BC application (Mehmood et al., 2020); on the other hand, it was also reported that BC supply led to a substantial increase in APX, MDHAR, DHAR, and GR activity in jute plants growing under SS (Hasanuzzaman et al., 2021). Sofy et al. (2021) also found that BC supplementation improves the activities of SOD, CAT, and POD, reducing ROS accumulation under SS. Moreover, the BC amendment also improved the Gly-1 and Gly-II activity in SS plants, which therefore reduced the MG-induced cellular damage to the plant growing under SS (Hasanuzzaman et al., 2021). Thus, BC assisted in an increase in antioxidant activities to protect the plant from salinity-induced deleterious impacts, therefore improving plant performance under SS.

## Biochar improves genes expression and stress responsive proteins under salinity stress

BC application also improves gene expression to induce salt tolerance. For instance, a BC-mediated increase in biomass and carbon assimilation in wheat plants under SS was linked with the up-regulation of water transporter genes (Soliman et al., 2022). Aquaporins play an imperative role in water transportation, particularly PIPs; they not only improve water uptake and transportation to roots and leaves (Cui et al., 2021), but also boost CO_2_ diffusion that ensures abundant substrate supply for photosynthesis and carbohydrate assimilation (Groszmann et al., 2017). It has been reported that BC in combination with Se-NPs upregulates the aquaporin and ion transporter genes, which regulate ionic homeostasis under SS (Santander et al., 2021). Moreover, BC + Se-NP mediated increase in Ca^2+^ concentration induced salinity tolerance by modifying cellular signaling pathways (Qin et al., 2019).

It has also been recorded that BC + Se-NPs increase the K**^+^**/Na**^+^** ratio and Ca^2+^ concentration *via* the Ca^2+^dependent SOS pathway. Similarly, ion transport proteins such as NHX1, HKT1, and SOS1 also play a key role in regulating salinity tolerance (Assaha et al., 2017; Li et al., 2022), and it has been reported that BC in combination with Se-NPs improved the expression of these genes, leading to a significant increase in salinity tolerance (Soliman et al., 2022). The findings of these authors also suggest a synergy between BC and Se-NPs for stabilizing membrane potential for optimal functioning of H + ATPase, favoring K uptake, and protecting wheat plants from salt-induced injury (Soliman et al., 2022). Further, SS also upregulates AQPs (P1P1, NIP, and N1P1), and these proteins are further upregulated by BC + Se-NPs, which promote water status equilibrium in plant cells and adjust their position to ROS-induced membrane damage (Soliman et al., 2022).

## Biochar maintains osmolytes accumulation and hormonal balance under salinity stress

Secondary metabolites, i.e., phenols and flavonoids, play an important role in ROS scavenging by improving antioxidant activities (Austen et al., 2019). According to Soliman et al. (2022), BC application markedly improved the concentration of phenols and flavonoids in salinity-stressed plants, with a parallel increase in the accumulation of different osmolytes (glycine betaine, proline, and carbohydrates). The BC-mediated increase in phenols and flavonoids strengthened the antioxidant defense system that prevents salinity-induced oxidative damage (Soliman et al., 2022). Some studies also documented that BC decreased the SS in plants and lowered the production of some hormones (Lashari et al., 2015; Akhtar et al., 2015a). For instance, BC application decreased the ABA contents of maize leaf sap growing under SS conditions (Lashari et al., 2015). In another study, authors also noted that BC application alone or in combination with entophytic bacteria decreased the xylem ABA contents of maize and wheat plants growing under SS as compared to controls (Akhtar et al., 2015b). The improvement in soil properties, i.e., soil moisture and Na^+^ binding in BC-amended soil decreases the root’s sensitivity to osmotic stress (Akhtar et al., 2015b). The incorporation of BC into salt-affected soils mitigates the effects of SS by decreasing Na^+^ uptake, which leads to a reduction in the ABA contents of cabbage plants grown under SS (Farhangi-Abriz and Torabian, 2018). BC application also increases the concentration of osmolytes that protect the plants from the damaging effects of SS. For instance, BC-mediated increase in GB and proline results in better osmotic adjustments under SS owing to the upregulation of antioxidant activity (Rasheed et al., 2019). However, some authors also noted that BC application reduced the accumulation of osmolytes. For instance, BC addition leads to a significant decrease in osmolytes accumulation owing to a reduction in exchangeable sodium under SS (Ghassemi-Golezani et al., 2020). Further BC treatments decrease the ABA, SA, and JA contents by decreasing the Na^+^ uptake and accumulation in plants (Ghassemi-Golezani and Rahimzadeh, 2022). Conversely, BC application increased the IAA synthesis by improving plant nutrient (Zn) uptake, owing to the fact Zn triggers tryptophan synthesis which is an essential amino acid for IAA production (Castillo-González et al., 2018). The reduction in ABA concentration following BC application substantially mitigated the adverse effects of SS (Ghassemi-Golezani and Rahimzadeh, 2022). The BC application improves water uptake and turgor pressure, which cause a reduction in ABA concentration (Ghassemi-Golezani and Rahimzadeh, 2022). The endogenous JA and SA are stress hormones, and they are directly linked with sodium concentration in plant parts (Ryu and Cho, 2015). Thus, BC has the potential to reduce oxidative stress and, therefore, reduce the synthesis of these hormones under SS (Ghassemi-Golezani and Rahimzadeh, 2022). In conclusion, BC maintains the accumulation of favorable hormones and osmolytes that protect plants from the toxic effects of SS and ensures better plant performance under SS.

## Biochar nutrition improves plant growth, yield, and quality under salinity stress

BC improves the growth and quality of plants grown under SS through various mechanisms (Akhtar et al., 2015c). BC amendment increased maize growth as well as biomass yield in soils containing a high concentration of Na^+^ and exchangeable salts (Kim et al., 2016). Likewise, BC supplementation improved the growth and biomass of tomatoes under SS (3.6 dS m^−1^) as compared to control conditions (Usman et al., 2016). In a field experiment, Lashari et al. (2013) found that BC application for six weeks in saline soil substantially improved the yield of wheat as compared to the control, whereas again in a 2-year field study, Lashari et al. (2015) found that BC amendments improved the plant height, leaf area, root density, photosynthesis, and grain yield under SS (Lashari et al., 2015).

Akhtar et al, 2015b and Akhtar et al, 2015c found that BC application improved the photosynthetic rate, root and shoot growth, leaf area, and yield of maize and wheat plants under SS as compared to the control treatment. Similarly, other authors also reported the same trend of increased in plant growth and biomass with BC application in saline-sodic soil (Lashari et al., 2015; Akhtar et al., 2015b). BC-mediated increase in growth and yield under SS is linked with improved soil physio-chemical and biological properties, improved CEC, nutrient and water uptake, microbial population, and a reduction in the uptake of Na^+^ (Akhtar et al., 2015a; Farhangi-Abriz and Torabian, 2018; Mansoor et al., 2021). BC-mediated increase in mineral and nutrient assimilation regulate chlorophyll synthesis, photosynthesis, and stress tolerance, which in turn improve plant growth (Soliman et al., 2022).

Soliman et al (2022) found that an increase in the growth of beans following BC application is linked with an improvement in soil pH and nutrient availability. Other authors also found that BC amendment (3%) increased nutrient concentration, which is the main factor responsible for the increase in plant growth; further, BC-mediated reduction in H_2_O_2_ production also leads to a substantial increase in plant growth under SS (Kaya et al., 2018). BC application has no impact on the essential oil contents of inflorescences and seeds in saline soils; however, an increase in the oil content of vegetative plant parts was observed with the application of BC (Ghassemi-Golezani and Rahimzadeh, 2022). Other authors also found that BC application improves essential oil contents by increasing nutrient availability to plants (Yadegari, 2017). Salinity stress modifies the composition of borage oil; however, BC + melatonin application appreciably increased palmitoleic acid (0.24%), stearic acid (4.01%), oleic acid (19.58%), linoleic acid (36.16%), α-linolenic-ω6 acid (19.52%), α-linolenic-ω3 acid (0.17%), and arachidic acid (0.27 wt.%) as compared to the control treatment (Farouk and Al-Huqail, 2022). Thus, a BC-mediated increase in plant performance is linked with an increase in soil properties, membrane stability, and antioxidant activities.

## Biochar improves soil properties to induce salinity tolerance

BC application plays an appreciable role in nutrient homeostasis under SS, and it has been recorded that BC application markedly decreased Na^+^ concentrations in potato xylem sap while BC application increased the K^+^ in xylem sap (Lashari et al., 2015; Akhtar et al., 2015a). Similarly, in another study, BC application reduced the Na^+^ uptake in lettuce and maize and increased the K uptake and accumulation (Hammer et al., 2015; Kim et al., 2016). Likewise, BC also increased K, N, and P contents and decreased the Na and Na^+^/K ratios of maize xylem sap (Lashari et al., 2015). BC application is very effective in reducing the Na^+^ uptake, and salt-affected soils can be cultivated by adapting the application of BC (Ding et al., 2020).

The use of organic amendments has emerged as an excellent tool to improve plant growth by changing the soil’s physio-chemical properties. Recently, the addition of BC to salt-affected soils has gained considerable attention across the globe (Amini et al., 2016; Ding et al., 2020). The application of BC to saline increases nutrient concentration owing to a concomitant increase in CEC, surface area, structure, porosity, and stability of soil structure (Zheng et al., 2018a). In salt-affected soils, higher Na^+^ concentration impairs K^+^ uptake; however, BC application significantly improved the K^+^ uptake as compared to the control (Lin et al., 2015). Biochar application also improves the NUE of crops owing to its porous structure and large surface area, which are conducive to an increase in NH^4+^ and a reduction in microbial de-nitrification (Liu et al., 2019).

Researchers have reported that BC with a high pH (9.6–10.8) increased the NH_3_ volatilization from salt-affected soil (Esfandbod et al., 2017; Sun et al., 2017); therefore, BC application effectively reduces the NH_3_ losses from saline and sodic soils. P availability is higher at pH 5.5–7; however, at pH >7, P availability substantially decreases. Nonetheless, BC addition increased the P availability in saline soils because of its inherent P fertilizer value and increased the growth of favorable bacteria (*Flavobacterium*, *Pseudomonas*, and *Thiobacillus*) that solubilize the unavailable P in soils (Ding et al., 2020). Similarly, BC application also improved nutrient availability, soil quality, and soil organic matter. All the studied BC rates increased the soil organic carbon (SOC); however, the application of 2.5% BC significantly improved the SOC by 10% as compared to other rates (Abo-Elyousr et al., 2022). Similarly, BC application also improved dehydrogenase activity, enhanced soil microbial biomass and organic matter stability, and led to a significant increase in nutrient absorption in saline soil (Abo-Elyousr et al., 2022).

## Biochar as an important amendment to improve soil fertility

**Figure 4:**
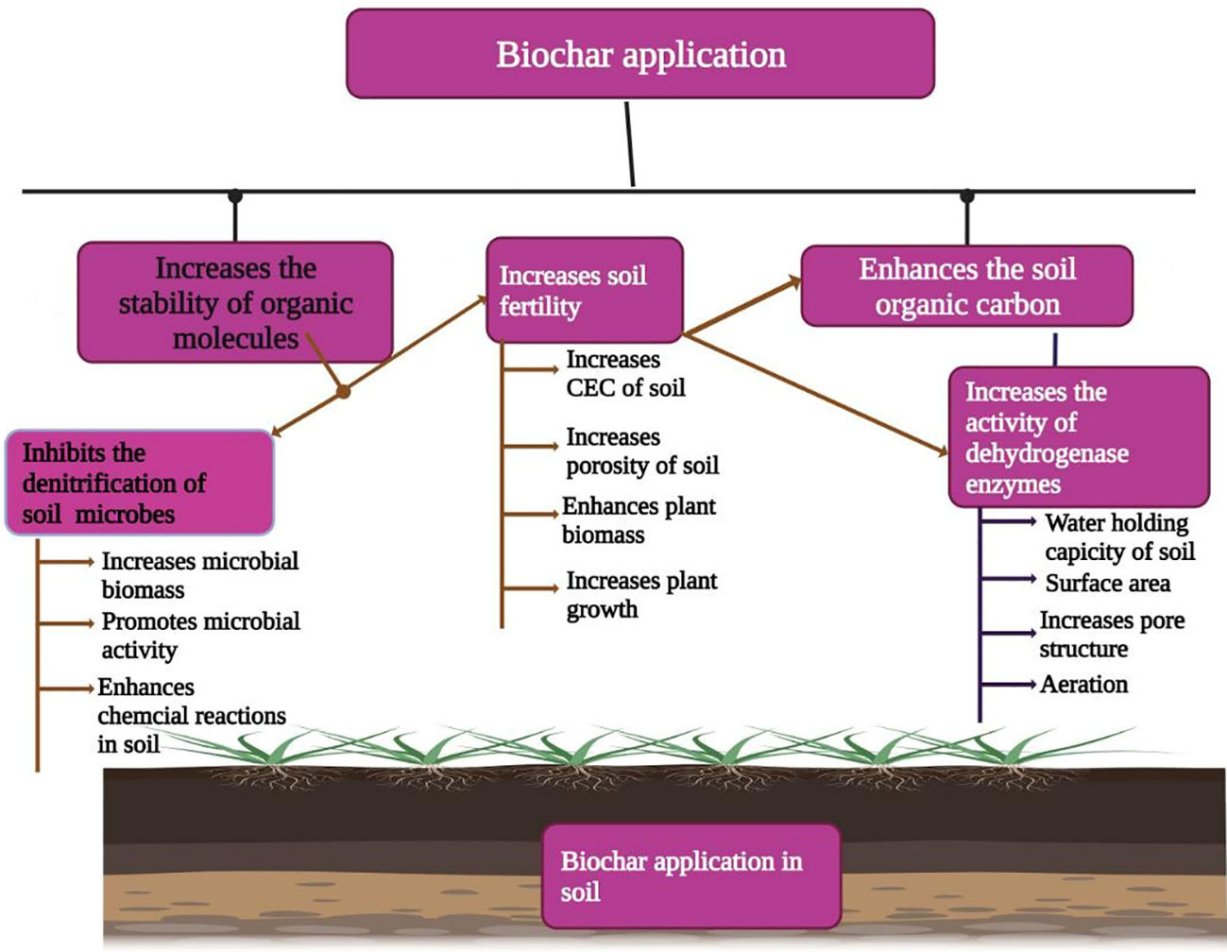
Biochar application improves soil fertility by increasing aggregate stability, microbial biomass, microbial activity, soil CEC, soil porosity, soil organic carbon, water holding capacity, and soil aeration.

Biochar application has been reported to improve soil fertility by incrassating soil pH, WHC, and CEC, retaining soil nutrients, and stimulating the activity of beneficial bacteria and fungi (Warnock et al., 2007). The incorporation of BC into soil alters soil properties including soil texture, structure, pore size, WHC soil bulk density, soil pore volume soil, soil porosity, WHC, and saturated hydraulic conductivity and water retention in soil (**Figure 4**, Downie et al., 2009; Abel et al., 2013; Zhang et al., 2017; Adekiya et al., 2020; Kapoor et al., 2022). The soil’s physical as well as hydraulic properties, directly and indirectly, affect the services provided by soil. For example, these properties also include root growth, soil aeration, soil compaction, and nutrient and water uptake. Biochar has also been reported to increase soil pH, soil organic carbon, CEC, and NUE and lead to an appreciable increase in plant growth after BC application (Van Zwieten et al., 2010; Agegnehu et al., 2017). Yamato et al. (2006) also noted that BC made from *Acacia magnum* increased soil pH, soil calcium, CEC, and base saturation, and according to Novak et al. (2009), BC application to acidic coastal soil substantially increased the soil pH, SOM, soil Ca, and Mg concentrations while BC decreased soil sulfur and Zn concentrations.

Researchers have also documented that BC did not equally increase the soil porosity when applied at the same rate due to the difference in soil type and soil class (Alghamdi, 2018). Generally, BC appreciably improved the soil porosity of coarse-textured soils as compared to fine-textured soils (Alghamdi, 2018). BC particles have porosity and intra-pore space that provide additional space for water retention; therefore, the addition of BC (30 Mg ha^−1^) significantly increased the moisture contents and water infiltration (Adekiya et al., 2020). However, improvement in soil moisture is linked with the rate of BC application (Kätterer et al., 2019).

El-Naggar et al. (2018) found that BC made from rice straw and crop residues substantially improved SOM, whereas Adekiya et al. (2020) found that BC made from hardwood (30 Mg ha^−1^) substantially improved SOM by 18% as compared to the unamended control. In another study, Yang and Lu (2020) noted a substantial increase in SOM after applying BC to rice as well as rapeseed straws. In a series of lab studies, a maximum decrease of 31% in soil BD was reported after BC application in sandy soil, whereas in coarse and fine-texture soils, BD was decreased by 14.2% and 9.2%, respectively, following BC application (Blanco-Canqui, 2017; Liu et al., 2017). El-Naggar et al. (2018) noted a sharp increase in soil pH following the application of BC made from umbrella tree residues and rice straw, respectively. Moreover, Chathurika et al. (2016) amended soil with 1% and 2% acer woodchip BC and found a significant increase in soil pH after 75 days as compared to control un-amended soil.

Zhang et al. (2017) noted an increase of 21% in CEC after BC application, whereas El-Naggar et al. (2018) found that BC made from rice straw, silver-grass residues, and umbrella trees to sandy soils increased the CEC by 906, 180, and 130%, respectively. Ndor et al. (2015) also found an increase of 21% in CEC after the application of 5 Mg ha^−1^ made from rice husk and sawdust, whereas they found an increase of 44% and 57% in CEC after the application of BC at rates of 10 Mg ha^−1^. Rodríguez-Vila et al. (2016) and Adekiya et al. (2020) found a substantial increase in soil N, P, K, S, Ca, and Mg status following BC application, whereas Yao et al. (2017) found a substantial increase in soil N concentration and decrease in total soil P after addition of maize straw BC (50–200 Mg ha^−1^). BC structure provides a refuge for beneficial microbes (bacteria and AMF), and it increases soil enzymatic activities. For instance, Demisie et al. (2014) found that BC application improved the activity of urease and b- glucosidase as compared to the control. Likewise, BC also activates the *Rhizobium*, which in turn increases the nodulation and nitrogenase activity and colonizes *Azotobactor* and *Azospirillum* (Gabhane et al., 2020). Karimi et al. (2020) observed a substantial increase in SMB after BC application, whereas Yao et al. (2017) noted an increase from 6.6% to 31.2% in fungal abundance after maize stalk BC application (50, 100, and 200 Mg ha^−1^) as compared to control.

## Biochar as an important amendment to improve crop growth and yield

Many authors have reported that BC application significantly improved the growth as well as biomass of different plant species (Seleiman et al., 2019). For example, Khan et al. (2017) found an increase of 49% in rapeseed yield following BC addition, whereas Rafique et al. (2020) noted that soil amended with BC showed an increase of 50%–55% in maize fresh and dry weights. Similarly, in sunflowers, BC improved growth and oil yield under water deficit conditions (Seleiman et al., 2019), while Zhang et al. (2020) noted higher cotton physiological activity in cotton plants amended with BC. Raboin et al. (2016) noted that maize yield in acid soil increased yield from 48% to 56% after the application of BC at 50 Mg ha^−1^. Agegnehu et al. (2015) also found an increase of 22% and 24% in seed and pod yield of peanuts after the application of BC (25 Mg ha^−1^) with inorganic fertilizers as compared to the control, while according to Xu et al. (2015), BC application increased the kernel quality of peanut.

Palansooriya et al., 2019 found a substantial increase in sweet potato yield after BC application, whereas, Gholizadeh et al. (2020) found no differences in yield between BC amended plots and control plots. Also, Mclennon et al. (2020) found that BC in combination with N fertilizer had no significant impacts on the growth and biomass productivity of *Schedonorus arundinacea* and *Poa pratensis* plants. These contradictory results could be due to differences in the physiochemical properties of BC. For instance, BC produced at a pyrolysis temperature of ≥600°C can absorb plant nutrients, therefore decreasing nutrient uptake. Moreover, Agegnehu et al. (2015) noted an increase in maize yield from 98% to 150% due to a parallel increase in WUE between 91% and 139%. Generally, increased crop yields and nutrient uptake might be due to the direct addition of nutrients from applied BC, which therefore increases the crop yield. Though crop responses to BC largely depend on BC type, soil type, and plant species. Asai et al. (2009) studied the impact of BC on the grain yield of rice grown in northern Laos, and they found a double increase in rice yield following BC application at 8 t ha^−1^. The experiments conducted by Abd-Elwahed et al. (2019) found that BC application (300 mg/L) enhanced wheat yield. Moreover, Salama et al. (2021) found the highest yields of 2.04 and 2.01 t ha^−1^ following the application of BC.

## Future implications

The use of biochar can reduce the negative impacts of abiotic stress depending on its biomass properties, feedstock type, and processing conditions. Biochar can be used to absorb air and water pollutants and salts, and it can also be used as a soil conditioner, compost additive, and carbon sequestration source to mitigate the adverse effects of climate change. Besides this, biochar can also contribute to the circular economy through its use in agriculture and horticulture. Moreover, different benefits of biochar have an appreciable potential for emerging bioenergy production systems. As the application of biochar has significantly increased in recent times, there is a need to develop guidelines and standards to produce this black gold. For instance, crop productivity can be decreased or increased by BC application depending on fertilizer management and soil type, and the chemical attitude of BC is also inconsistent with heavy metals. The interaction mechanisms, productive technologies, applications, and properties between soil, plant, and biochar are very critical and yet not thoroughly discovered. Therefore, more efforts are needed to underpin all these things for the promising future of biochar.

## Conclusion

Drought and salinity stress induce serious alterations in plant growth and development by disturbing various biochemical, physiological, and molecular processes, BC application improves membrane stability, nutrient uptake, and nutrient homeostasis, thereby improving plant performance under drought and salinity stress. The BC amendment also improves photosynthetic efficiency and antioxidant activity, which maintains hormonal balance and protects the plant from drought and salinity-induced oxidative and osmotic stresses and improves plant performance. In the case of salinity stress, BC also restricted the entry of noxious Na^+^ and increased the entry of K^+^, which regulates stomata movements and improves the leaf gas exchange characteristics under salinity stress.

Yet many unanswered questions exist regarding the role of BC in different plant processes under drought and salinity stresses. For instance, the role of seed germination under both stresses has not been studied yet; therefore, future studies must be conducted to determine the role of BC in the different mechanisms involved in seed germination. More research studies are required to explore the effect of BC on nutrient signaling *via* ionic transporters and nutrient channels under both stresses. The role of BC in protecting the photosynthetic apparatus from oxidative stress and the effect of BC on stomatal signaling and the regulation of guard cells must be explored. The role of BC on plant reproductive characteristics hass not been studied yet; therefore, it is crucial to study the effect of BC on this aspect under both stresses. The role of BC on hormones and osmolytes accumulation is poorly studied, so it is mandatory to explore the role of BC in the accumulation of different osmolytes and hormones under drought and salinity stress. It would also be fascinating to determine the effect of BC on the complex relationship between salicylic acid, IAA, gibberellic acid, cytokinin, and ethylene at the transcriptomic level.

The role of BC under drought and salinity stresses is less studied in field conditions; therefore, it is suggested to conduct long-term field studies under a wide range of climate conditions to increase understanding about the role of BC in mediating drought as well as salinity stresses. The use of BC in combination with microbes would enhance the plant’s tolerance to both stresses. However, detailed studies are needed to determine the effectiveness of BC and microbes in improving growth under water deficiency and salinity stress. Moreover, pilot scale studies are also needed to develop models to recommend the rates of BC application based on soil, plant, and climatic conditions.

## Author contributions

YW and YZ: conceptualization. YW, LZ, and XW: writing original draft. XW, YZha, and XL: writing-review and editing. All authors have read and agreed to the published version of the manuscript.
